# Association of visual display terminal time with prevalence of temporomandibular disorder among Japanese workers

**DOI:** 10.1002/1348-9585.12370

**Published:** 2022-11-09

**Authors:** Takashi Zaitsu, Yuko Inoue, Akiko Oshiro, Akira Nishiyama, Yoko Kawaguchi, Jun Aida

**Affiliations:** ^1^ Department of Oral Health Promotion, Graduate School of Medical and Dental Science Tokyo Medical and Dental University Tokyo Japan; ^2^ Department of General Dentistry, Graduate School of Medical and Dental Science Tokyo Medical and Dental University Tokyo Japan

**Keywords:** temporomandibular disorders (TMD), temporomandibular disorders related symptoms (TRS), visual display terminal (VDT)

## Abstract

**Objectives:**

Visual display terminal (VDT) time has been reported to affect the development of temporomandibular disorders (TMDs). However, no study has investigated the association between VDT time at and outside of work with TMDs Adjusting for known TMJ risk factors. This study aimed to investigate whether TMDs were associated with VDT time at and outside of work after adjusting for various working conditions in Japanese workers.

**Methods:**

This cross‐sectional study was based on an internet survey of 3930 workers (2057 men and 1873 women), The TMD Screening Questionnaire (SQ‐TMD), occupational factors, VDT time at and outside of work, psychosocial factors, and habits were assessed. We applied logistic regression to estimate the odds ratio (OR) of VDT time on SQ‐TMD with adjustment for confounders.

**Results:**

The mean age of the respondents was 43.3 ± 11.7 years, and 778 (19.8%) and 3152 (80.2%) subjects were at high and low TMD‐related symptoms (TRS). Logistic regression analysis adjusting for all covariates (Model 2), the prevalence of high TRS was significantly higher among those with VDT time at work of 60–179 min (OR = 1.52, 95% CI 1.18–1.94), 180–359 min (OR = 1.27, 95% CI 1.00–1.62), and more than 360 min (OR = 1.44, 95% CI 1.10–1.88) compared to those with 0–59 min. However, there was no significant difference in the prevalence of high TRS for VDT time outside of work.

**Conclusion:**

VDT time at work, but not VDT time outside of work, influences the prevalence of TRS. Since the association between VDT time at work and the prevalence of TRS was found even after adjusting for sociopsychological factors and habits generally associated with TMD, further investigation of other factors is needed.

## 1 INTRODUCTION

Temporomandibular disorders (TMDs) and their related symptoms are prevalent health conditions among workers.^1^ TMDs are disorders that produce symptoms in the temporomandibular joint or masticatory muscles and are multifactorial disorders manifested by multiple factors. Studies have reported the prevalence of TMDs and related symptoms among workers and the general population as 17–18% and 5–12%, respectively.^2^ In spite of the burden of TMD, there is currently insufficient research on the risk factors associated with the development of TMD. TMD is composed of more than 30 health problems related to the temporomandibular joints and the muscles and tissues of the jaw, with a wide range of causes.^3^ Although psychosocial factors such as stress and habits such as teeth grinding and tooth contact habit have been reported as risk factors of TMDs in general, other factors have not been sufficiently investigated.

Working environments and workers' body posture during work are considered to affect the risk of TMD.^2^ Recently, many workers spend significant time with a visual display terminal (VDT). Therefore, its health effects have attracted attention.^3^, ^4^ A “VDT” is a generic term for monitors and tablets associated with personal computers (PCs). Long‐term engagement in VDT tasks causes physical and mental problems.^5^, ^6^, ^7^, ^8^ An increase in the duration of PC use is also associated with the risk of TMD.^9^ A previous study reported that each 2‐h increase in PC use was associated with the presence of TMD.

However, no study has examined the association of both VDT time at and outside of work with TMD. Nowadays, an increasing number of people are using VDTs outside of work hours due to hobbies or other things. Therefore, the aim of this study was to investigate how VDT time at work and outside work affects TMDs. We hypothesized that an increase in VDT time at work and outside of work is associated with an increased risk of TMD.

## 2 METHODS

### 2.1 Study settings

This cross‐sectional study used data obtained from an internet‐based self‐reported questionnaire survey conducted in March 2017. The participants were initially recruited through services managed by company M. At the time of registration, participants were asked to provide written consent. The participants in this study were recruited for the purpose of covering all occupations and to gather a total of 4000 people of all 11 occupational categories of the Ministry of Health, Labor and Welfare, with equal numbers of men and women. Company M boasts the No. 1 track record in Japan in online research, and although the target audience for Company M's monitors is limited to those aged 6 and older, it encompasses a wide range of subjects. Three thousand nine hundred and thirty (2057 males and 1873 females) workers (mean age, 43.3 ± 11.7 years) were recruited within the recruitment deadline.

### 2.2 Dependent variable

The prevalence of TMD was used as the dependent variable. It was measured by the Screening Questionnaire for TMD (SQ‐TMD), developed and validated by Sugizaki et al., which was used to screen the subjects for TMD‐related symptoms (TRS).^10^ The SQ‐TMD consists of four screening items related to TMS as follows: (a) limited mouth opening was assessed using the question “When your mouth is opened wide, are you able to insert three fingers lined up with the ring finger from the index finger longitudinally?” (b) pain during mouth opening was assessed using the question “Is there jaw pain when opening the mouth wide and closing tightly?” (c) deviation during the opening was assessed using the question “When the mouth is opened wide, is it straight?” and (d) pain during hard mastication was assessed using the question “Is there jaw or facial pain when eating hard things such as dry meat and octopus?” Each question was answered on a 5‐point scale. Based on the sum of the responses to the four items, the total score varied from 4 to 20; subjects scoring 9 points or more were included in the high‐TRS group, and those scoring 8 points or less were part of the low‐TRS group. This screening question did not include the joint sound question. This is because responses to the five questions that included the joint sound question and responses to the four questions that excluded this question were compared using nonparametric dichotomous item response theory, and responses using the four questions showed higher validity for TMJ disorder than responses using the five questions.^10^

### 2.3 Independent variables

VDT time at work and outside of work were used as independent variables. VDT time at work was assessed using the question “How long, on average, do you spend in front of a monitor (including PCs, tablets, and smartphones) at work?” VDT time outside of work was assessed using the question “How long, on average, do you spend in front of a monitor (including PCs, tablets, and smartphones) in a non‐work setting, such as at home?” The participants were asked to respond to these questions in hours and minutes. Each VDT time was calculated in minutes this time and classified into quadrants. As a result, VDT time (at work) was divided into 0–59 min, 60–179 min, 180–359 min, and 360 min or more. VDT time (outside of work) was also divided into 0–59 min, 60–119 min, 120–179 min, and 180 min or more.

### 2.4 Covariates

As covariates, we used sociodemographic information and worker characteristics that were considered strongly related to VDT: industry classification, occupational classification, and work shift. Psychosocial factors and habits related to TMJ disorder, the objective variable, were also used as covariates.

Sociodemographic information, industrial classification, occupational classification, and work shift were used as covariates. Sociodemographic information included age, sex, education (primary and secondary school graduation, high school graduation, professional school graduation, junior college graduation, university graduation, graduation from a Master's program, graduation from a doctoral program, and others), and individual annual income (<2 million yen, 2–3.9 million yen, 4–5.9 million yen, 6–7.9 million yen, 8–9.9 million yen, >10 million yen, and unknown/blank). The occupations of the participants were classified into the following 20 types according to the Japanese Standard Industrial Classification (revised October 2013) 12th revision^11^: (a) agriculture and forestry; (b) fisheries; (c) mining or quarrying stone; (d) construction industry; (e) manufacturing; (f) electricity, gas, heat supply, and water; (g) information and communications; (h) transportation and postal activities; (i) wholesale and retail trade; (j) finance and insurance; (k) real estate and goods rental, (l) scientific research, and technical services; (m) accommodation and eating services; (n) life‐related, entertainment, and recreation services; (o) education and learning support; (p) healthcare and welfare; (q) compound services; (r) services (not otherwise classified); (s) public duties (excluding those classified elsewhere); and (t) industries that cannot be classified. Of these, we defined classes 1 to 2 as “primary industry,” 3 to 5 as “secondary industry,” and 6 to 20 as “tertiary industries.” Workers' occupations were classified into the following 11 occupations based on the Japanese Standard Occupational Classification (December 2009 Statistical Criteria Setting Classification)^12^: (a) administrative professionals (company officers, company administrative officers, public administrative officers, etc.); (b) professional and technical professionals (research, health, faculty, etc.); (c) office workers (human affairs, labor, accounting, management, etc.); (d) marketing personnel (sales, etc.); (e) service professionals (facilities and equipment management, custom centers, home helpers, beauty technicians, etc.); (f) security occupational workers (self‐defense officers, police officers, security officers, etc.); (g) agricultural, forestry, and fishery workers (landscapers, fishermen, officers, etc.); (h) production process workers (steel maintenance control and monitoring workers, gum and plastic product manufacturing workers, etc.); (i) transportation and machinery drivers (taxis, bus drivers, palms, etc.); (j) construction and mining workers (carpenter, plumbing, civil engineers, etc.); and (k) personnel involved in transportation, cleaning, packaging, etc. (delivery personnel, cleaning personnel, etc.). Of these, we defined classes 1 to 4 as “white collar” and 5 to 11 as “blue collar.”^13^ Workers' work shifts were classified into day shifts, night shifts, both day and night shifts, and flexes and other work shifts.

We considered psychosocial factors and habits as known risk factors of TMD. Psychosocial factors related to TMD (stress, anxiety, depression, and fatigue) were assessed using the following questions^1^: (a) “Is there stress at work, school, home, or in your personal relationships? (stress)”; (b) “Do you have anxiety at work, school, home, or personal relationships? (anxiety)”; (c) “Is there a feeling of depression or actual depression? (depression)”; and (d) “Do you feel fatigued or restless? (tired)” The items of habits related to TMD were assessed as follows: (a) “Are the upper and lower teeth in contact when working at a desk or when concentrating and doing something?” (tooth contact habit [TCH])^14^; (b) “Did you experience tooth grinding during sleep in the last 3 months?” (bruxism)^15^; and (c) “Is there a sensation of rubbing teeth when awake? (clenching).^15^ Each question was answered on a 5‐point scale: “never,” “occasionally,” “Difficult to say,” “often,” or “always.” We classified “never,” “occasionally,” and “difficult to say” as “None,” and “often” or “always” as “Present.”

### 2.5 Statistical analyses

The preferences of SQ‐TMD (high‐TRS group) and VDT time (at work and outside of work) Quartile by age, sex, education, individual annual income, industrial classification, occupational classification, work shifts, psychosocial factors, and habits related to TMD are shown as descriptive statistics with chi‐square test. Logistic regression analysis was performed with the two values of SQ‐TMD as the dependent variable and VDT time on the job and outside of work hours as the independent variable. To examine the impact of psychological and behavioral mediators, we built multiple models. First, univariate analyses were conducted (Model 1). Then, variables of VDT time and all covariates were included in the model (Model 2). The at‐work and outside‐of‐work VDT time variables are put into the model together. This is because the correlation coefficient between the two is as low as 0.102 on Spearman's correlation coefficient, and the two types of time are considered to be related to TMJ independently of each other. Statistical analysis was performed using SPSS version 20.0 (IBM, Armonk, NY), and the significance level was set at 5%.

## 3 RESULTS

The mean age of the participants in this study was 43.3 ± 11.7 years. The sex ratio of the participants was 2057 (52.3%) males and 1873 (47.7%) females. Among the participants, 778 (19.8%) and 3152 (80.2%) subjects were at high and low TRS, respectively. Table 1 shows the association between the basic characteristics and SQ‐TMD (high‐TRS group). The results show that the high‐TRS group was higher among young people, those in the secondary and tertiary industries, and those with all psychosocial factors and habits except the tooth contact habit. For VDT time, there was a significant association between VDT time at work and SQ‐TMD, but no significant association was found between VDT time outside of work and SQ‐TMD.

**TABLE 1:** Descriptive distribution of screening questionnaire‐for temporomandibular disorders high‐TSR group (*N* = 3930)

			SQ‐TMD*				
		N	high‐TSR group		low‐TSR group		*p*‐value
			N(%)		N(%)		
Age (years)	20s	625	139(22.2)		486(77.8)		**<0.001**
	30s	969	205(21.2)		764(78.8)		
	40s	1011	220(21.8)		791(78.2)		
	50s	888	158(17.8)		730(82.2)		
	60 years or older	437	56(12.8)		381(87.2)		
Sex	Male	2057	399(19.4)		1658(80.6)		0.510
	Female	1873	379(20.2)		1494(79.8)		
Education (graduation)	Primary and secondary school	110	27(24.5)		83(75.5)		0.806
	High school	1306	266(20.4)		1040(79.6)		
	Junior college	527	101(19.2)		426(80.8)		
	Vocational school	312	55(17.6)		257(82.4)		
	University	1521	300(19.7)		1221(80.3)		
	Master's or doctorial program	142	27(19.0)		115(81.0)		
	Others	12	2(16.7)		10(83.3)		
Individual annual income	<2 million yen	577	122(21.1)		455(78.9)		0.713
	2–3.9 million yen	1479	299(20.2)		1180(79.8)		
	4–5.9 million yen	810	153(18.9)		657(81.1)		
	6–7.9 million yen	299	51(17.1)		248(82.9)		
	8–9.9 million yen	134	24(17.9)		110(82.1)		
	≧10 million yen	76	13(17.1)		63(82.9)		
	Unknown/blank	555	116(20.9)		439(79.1)		
Industrial	Primary industry	305	43(14.1)		262(85.9)		**0.021**
Classification	Secondary Industry	1097	233(21.2)		864(78.8)		
	Tertiary industry	2528	502(19.9)		2026(80.1)		
Occupational	White collar	1122	206(18.4)		916(81.6)		0.153
Classification	Blue collar	2808	572(20.4)		2236(79.6)		
Work shifts	Day shift	3155	633(20.1)		2522(79.9)		0.098
	Both day and night shifts	87	24(27.6)		63(72.4)		
	Night shift	489	90(18.4)		399(81.6)		
	Flexes, etc.	199	31(15.6)		168(84.4)		
**Psychosocial factors**							
Stress	None	2543	466(18.3)		2077(81.7)		**0.002**
	Present	1387	312(22.5)		1075(77.5)		
Anxiety	None	2743	491(17.9)		2252(82.1)		**<0.001**
	Present	1187	287(24.2)		900(75.8)		
Depression	None	2986	527(17.6)		2459(82.4)		**<0.001**
	Present	944	251(26.6)		693(73.4)		
Feel tired	None	2797	490(17.5)		2307(82.5)		**<0.001**
	Present	1133	288(25.4)		845(74.6)		
**Habit**							
Tooth contact habit	None	3042	592(19.5)		2450(80.5)		0.329
	Present	888	186(20.9)		702(79.1)		
Bruxism	None	3607	666(18.5)		2941(81.5)		**<0.001**
	Present	323	112(34.7)		211(65.3)		
Clenching	None	3601	658(18.3)		2943(81.7)		**<0.001**
	Present	329	120(36.5)		209(63.5)		
Visual display terminal time (at work)	0–59 min	929	154(16.6)		775(83.4)		**0.005**
	60–179 min	887	192(21.6)		695(78.4)		
	180–359 min	1102	251(19.0)		851(81.0)		
	≧360 min	1012	181(22.8)		831(77.2)		
Visual display terminal time	0‐60 min	1082	66(19.4)		1016(80.6)		0.071
(outside of work)	61–120 min	1180	372(19.4)		808(80.6)		
	121–180 min	827	148(17.9)		679(82.1)		
	≧181 min	841	192(22.8)		649(77.2)		

Table 2 shows the relationship between the basic characteristics and VDT time at work. The results showed significant associations with all items except for the tooth contact habit. Table 3 shows the association between the basic characteristics and VDT time outside of work. Significant associations were found for age, personal income, occupational classification, all psychosocial factors, and bruxism.

**TABLE 2:** Descriptive distribution of visual display terminal time at work (Quartile) (*N* = 3930)

		total	0–59 min	60–179 min	180–359 min	≧360 min	
		N	N(%)	N(%)	N(%)	N(%)	*p*‐value
Age (years)	20s	625	145(23.2)	125(20.0)	201(32.2)	154(24.6)	**<0.001**
	30s	969	220(22.7)	201(20.7)	311(32.1)	237(24.5)	
	40s	1011	253(25.0)	226(22.4)	316(31.3)	216(21.4)	
	50s	888	206(23.2)	211(23.8)	325(36.6)	146(16.4)	
	60 years or older	437	105(24.0)	124(28.4)	166(38.0)	42(9.6)	
Sex	Male	2057	502(24.4)	500(24.3)	714(34.7)	341(16.6)	**<0.001**
	Female	1873	427(22.8)	387(20.7)	605(32.3)	454(24.2)	
Education	Primary and secondary school	110	37(33.6)	37(33.6)	28(25.5)	8(7.3)	**<0.001**
(graduation)	High school	1306	462(35.4)	287(22.0)	374(28.6)	183(14.0)	
	Junior college	527	147(27.9)	146(27.7)	145(27.5)	89(16.9)	
	Vocational school	312	74(23.7)	68(21.8)	103(33.0)	67(21.5)	
	University	1521	200(13.1)	328(21.6)	599(39.4)	394(25.9)	
	Master's or doctorial program	142	7(4.9)	19(13.4)	67(47.2)	49(34.5)	
	Others	12	2(16.7)	2(16.7)	3(25.0)	5(41.7)	
Individual annual income	<2 million yen	577	202(35.0)	118(20.5)	181(31.4)	76(13.2)	**<0.001**
	2–3.9 million yen	1479	409(27.7)	359(24.3)	419(28.3)	292(19.7)	
	4–5.9 million yen	810	145(17.9)	185(22.8)	306(37.8)	174(21.5)	
	6–7.9 million yen	299	25(8.4)	69(23.1)	128(42.8)	77(25.8)	
	8–9.9 million yen	134	8(6.0)	20(14.9)	79(59.0)	27(20.1)	
	≧10 million yen	76	2(2.6)	9(11.8)	47(61.8)	18(23.7)	
	Unknown/blank	555	138(24.9)	127(22.9)	159(28.6)	131(23.6)	
Industrial	Primary industry	305	130(42.6)	98(32.1)	61(20.0)	16(5.2)	**<0.001**
Classification	Secondary Industry	1097	271(24.7)	191(17.4)	372(33.9)	263(24.0)	
	Tertiary industry	2528	528(20.9)	598(23.7)	886(35.0)	516(20.4)	
Occupational	White collar	1122	57(5.1)	153(13.6)	513(45.7)	399(35.6)	**<0.001**
Classification	Blue collar	2808	872(31.1)	734(26.1)	806(28.7)	396(14.1)	
Work shifts	Day shift	3155	688(21.8)	667(21.1)	1101(34.9)	699(22.2)	**<0.001**
	Both day and night shifts	87	35(40.2)	26(29.9)	21(24.1)	5(5.7)	
	Night shift	489	167(34.2)	150(30.7)	127(26.0)	45(9.2)	
	Flexes, etc.	199	39(19.6)	44(22.1)	70(35.2)	46(23.1)	
**Psychosocial factors**							
Stress	None	2543	598(23.5)	600(23.6)	898(35.3)	447(17.6)	**<0.001**
	Present	1387	331(23.9)	287(20.7)	421(30.4)	348(25.1)	
Anxiety	None	2743	648(23.6)	645(23.5)	953(34.7)	497(18.1)	**<0.001**
	Present	1187	281(23.7)	242(20.4)	366(30.8)	298(25.1)	
Depression	None	2986	700(23.4)	696(23.3)	1042(34.9)	548(18.4)	**<0.001**
	Present	944	229(24.3)	191(20.2)	277(29.3)	247(26.2)	
Feel tired	None	2797	658(23.5)	652(23.3)	994(35.5)	493(17.6)	**<0.001**
	Present	1133	271(23.9)	235(20.7)	325(28.7)	302(26.7)	
**Habit**							
Tooth contact habit	None	3042	704(23.1)	699(23.0)	1041(34.2)	598(19.7)	0.098
	Present	888	225(25.3)	188(21.2)	278(31.3)	197(22.2)	
Bruxism	None	3607	869(24.1)	819(22.7)	1214(33.7)	705(19.5)	**0.002**
	Present	323	60(18.6)	68(21.1)	105(32.5)	90(27.9)	
Clenching	None	3601	866(24.0)	824(22.9)	1213(33.7)	698(19.4)	**<0.001**
	Present	329	63(19.1)	63(19.1)	106(32.2)	97(29.5)	

**TABLE 3:** Descriptive distribution of visual display terminal time outside of work (Quartile) (*N* = 3930)

		total	0–60 min	61–120 min	121–180 min	≧181 min	
		N	N(%)	N(%)	N(%)	N(%)	p‐value
Age (years)	20 s	625	46(7.4)	258(41.3)	142(22.7)	179(28.6)	**<0.001**
	30 s	969	59(6.1)	488(50.4)	202(20.8)	220(22.7)	
	40 s	1011	94(9.3)	507(50.1)	203(20.1)	207(20.5)	
	50 s	888	90(10.1)	452(50.9)	185(20.8)	161(18.1)	
	60 years or older	437	51(11.7)	217(49.7)	95(21.7)	74(16.9)	
Sex	Male	2057	159(7.7)	1029(50.0)	432(21.0)	437(21.2)	0.145
	Female	1873	181(9.7)	893(47.7)	395(21.1)	404(21.6)	
Education	Primary and secondary school	110	7(6.4)	46(41.8)	25(22.7)	32(29.1)	0.054
(graduation)	High school	1306	122(9.3)	621(47.5)	271(20.8)	292(22.4)	
	Junior college	527	46(8.7)	259(49.1)	110(20.9)	112(21.3)	
	Vocational school	312	35(11.2)	144(46.2)	55(17.6)	78(25.0)	
	University	1521	122(8.0)	766(50.4)	342(22.5)	291(19.1)	
	Master's or doctorial program	142	7(4.9)	82(57.7)	21(14.8)	32(22.5)	
	Others	12	1(8.3)	4(33.3)	3(25.0)	4(33.3)	
Individual annual income	<2 million yen	577	67(11.6)	239(41.4)	127(22.0)	144(25.0)	**<0.001**
	2–3.9 million yen	1479	127(8.6)	712(48.1)	311(21.0)	329(22.2)	
	4–5.9 million yen	810	71(8.8)	412(50.9)	168(20.7)	159(19.6)	
	6–7.9 million yen	299	17(5.7)	167(55.9)	67(22.4)	48(16.1)	
	8–9.9 million yen	134	3(2.2)	85(63.4)	20(14.9)	26(19.4)	
	≧10 million yen	76	8(10.5)	45(59.2)	11(14.5)	12(15.8)	
	Unknown/blank	555	47(8.5)	262(47.2)	123(22.2)	123(22.2)	
Industrial	Primary industry	305	35(11.5)	137(44.9)	67(22.0)	66(21.6)	0.614
Classification	Secondary Industry	1097	95(8.7)	537(49.0)	228(20.8)	237(21.6)	
	Tertiary industry	2528	210(8.3)	1248(49.4)	532(21.0)	538(21.3)	
Occupational	White collar	1122	75(6.7)	619(55.2)	202(18.0)	226(20.1)	**<0.001**
Classification	Blue collar	2808	265(9.4)	1303(46.4)	625(22.3)	615(21.9)	
Work shifts	Day shift	3155	267(8.5)	1570(49.8)	660(20.9)	658(20.9)	0.078
	Both day and night shifts	87	6(6.9)	35(40.2)	15(17.2)	31(35.6)	
	Night shift	489	44(9.0)	226(46.2)	111(22.7)	108(22.1)	
	Flexes, etc.	199	23(11.6)	91(45.7)	41(20.6)	44(22.1)	
**Psychosocial factors**							
Stress	None	2543	234(9.2)	1293(50.8)	528(20.8)	488(19.2)	**<0.001**
	Present	1387	106(7.6)	629(45.3)	299(21.6)	353(25.5)	
Anxiety	None	2743	253(9.2)	1390(50.7)	562(20.5)	538(19.6)	**<0.001**
	Present	1187	87(7.3)	532(44.8)	265(22.3)	303(25.5)	
Depression	None	2986	269(9.0)	1529(51.2)	601(20.1)	587(19.7)	**<0.001**
	Present	944	71(7.5)	393(41.6)	226(23.9)	254(26.9)	
Feel tired	None	2797	259(9.3)	1408(50.3)	565(20.2)	565(20.2)	**<0.001**
	Present	1133	81(7.1)	514(45.4)	262(23.1)	276(24.4)	
**Habit**							
Tooth contact habit	None	3042	261(8.6)	1498(49.2)	654(21.5)	629(20.7)	0.171
	Present	888	79(8.9)	424(47.7)	173(19.5)	212(23.9)	
Bruxism	None	3607	325(9.0)	1768(49.0)	762(21.1)	752(20.8)	**0.005**
	Present	323	15(4.6)	154(47.7)	65(20.1)	89(27.6)	
Clenching	None	3601	322(8.9)	1767(49.1)	755(21.0)	757(21.0)	0.060
	Present	329	18(5.5)	155(47.1)	72(21.9)	84(25.5)	

Table 4 shows the results of multivariate logistic regression analysis of the association of VDT time and other variables with TMD prevalence. Univariate logistic regression analysis (Model 1) showed that VDT time at work was significantly associated with a higher prevalence of TMD for 60–179 min (OR = 1.39, 95% CI 1.10–1.76), and more than 360 min (OR = 1.48, 95% CI 1.17–1.89) compared to those with 0–59 min. For VDT time outside of work, no significant difference in the prevalence of TMD was observed. Adjusting for all covariates (Model 2), the prevalence of TMD was significantly higher among those with VDT time at work of 60–179 min (OR = 1.52, 95% CI 1.18–1.94), 180–359 min (OR = 1.27, 95% CI 1.00–1.62), and more than 360 min (OR = 1.44, 95% CI 1.10–1.88) compared to those with 0–59 min. However, there was no significant difference in the prevalence of TMD for VDT time outside of work.

**TABLE 4:** Logistic regression analysis of the association between VDT time and SQ‐TMD (*N* = 3930)

		Model 1						Model 2		
		VDT time (at work)			VDT time (outside of work)					
		OR	95%CI		OR	95%CI		OR	95%CI	
			Lower	Upper		Lower	Upper		Lower	Upper
VDT time (at work)	0–59 min (reference)									
	60–179 min	**1.39**	**1.10**	**1.76**	—	—	—	**1.52**	**1.18**	**1.94**
	180–359 min	1.18	0.95	1.48	—	—	—	**1.27**	**1.00**	**1.62**
	≧360 min	**1.48**	**1.17**	**1.89**	—	—	—	**1.44**	**1.10**	**1.88**
VDT time(outside of work)	0–59 min (reference)	—	—	—						
	60–119 min	—	—	—	1.00	0.74	1.33	0.87	0.64	1.17
	120–179 min	—	—	—	0.91	0.66	1.25	0.78	0.56	1.08
	≧180 min	—	—	—	**1.23**	**0.90**	**1.68**	1.02	0.74	1.41
Age	20s (reference)	—	—	—	—	—	—			
	30s	—	—	—	—	—	—	0.98	0.76	1.26
	40s	—	—	—	—	—	—	1.04	0.81	1.34
	50s	—	—	—	—	—	—	0.86	0.65	1.13
	60 years or older	—	—	—	—	—	—	**0.65**	**0.45**	**0.93**
Sex	male(reference)	—	—	—	—	—	—			
	Female	—	—	—	—	—	—	0.84	0.70	1.02
Education (graduation)	Primary ad secondary school(reference)	—	—	—	—	—	—			
	High school	—	—	—	—	—	—	0.90	0.57	1.44
	Junior college	—	—	—	—	—	—	0.79	0.48	1.30
	Vocational school	—	—	—	—	—	—	0.75	0.44	1.29
	University	—	—	—	—	—	—	0.87	0.54	1.39
	Masters program and Doctorial course	—	—	—	—	—	—	0.82	0.43	1.55
	Others	—	—	—	—	—	—	0.83	0.17	4.14
Individual annual income	<2 million yen (reference)	—	—	—	—	—	—			
	2–3.9 million yen	—	—	—	—	—	—	0.89	0.70	1.14
	4–5.9 million yen	—	—	—	—	—	—	0.80	0.60	1.08
	6–7.9 million yen	—	—	—	—	—	—	0.75	0.51	1.12
	8–9.9 million yen	—	—	—	—	—	—	0.87	0.51	1.47
	≧10 million yen	—	—	—	—	—	—	0.83	0.42	1.62
	Unknown/blank	—	—	—	—	—	—	0.94	0.70	1.26
Industrial Classification	Primary industry(reference)	—	—	—	—	—	—			
	Secondary Industry	—	—	—	—	—	—	**1.53**	**1.06**	**2.22**
	Tertiary industry	—	—	—	—	—	—	**1.45**	**1.01**	**2.06**
Occupational classification	White collar(reference)	—	—	—	—	—	—			
	Blue‐collar worker	—	—	—	—	—	—	1.13	0.92	1.40
Work shifts	Day shift(reference)	—	—	—	—	—	—			
	Both day and night shifts	—	—	—	—	—	—	1.52	0.92	2.50
	Night shift	—	—	—	—	—	—	0.87	0.67	1.13
	Flexes, etc.	—	—	—	—	—	—	0.78	0.52	1.17
Psychosocial factors	Stress	—	—	—	—	—	—	0.77	0.59	1.00
(reference = No)	Anxiety	—	—	—	—	—	—	1.15	0.86	1.54
	Depression	—	—	—	—	—	—	**1.34**	**1.02**	**1.77**
	Feel tired	—	—	—	—	—	—	1.23	0.98	1.54
Habit	Tooth contact habit	—	—	—	—	—	—	0.84	0.69	1.03
(reference = No)	Bruxism	—	—	—	—	—	—	**1.60**	**1.20**	**2.11**
	Clenching	—	—	—	—	—	—	**1.95**	**1.47**	**2.59**

*Note*: Model 1: Univariate analysis. Model 2: Multivariable analysis adjusted for all covariates.

Abbreviations: CI, confidence interval; OR, odds ratio; SQ‐TMD, TMD Screening Questionnaire; TMD, temporomandibular disorders; VDT, visual display terminal.

## 4 DISCUSSION

VDT time at work was positively associated with the prevalence of TMD. However, the VDT time outside of work did not show any significant association. However, after considering psychosocial factors and habits, it was still significantly associated with the prevalence of TMD.

The present results are consistent with those of previous studies. First, in relation to the prevalence of TMD prevalence, there was no major difference in the proportion of workers with TMDs in this study compared with previous studies. In this study, 19.4% of males, 20.2% of females, and 19.8% of the total population were in the high‐TRS group. In previous studies, involving workers using the same questionnaire, approximately 18% of the population in the study by Sugizaki et al.^1^ and 16.4% in the study by Nishiyama et al.^16^ were at high TRS. In addition, in the current study, the prevalence of TMD was higher in those with longer VDT time at work compared to those with shorter VDT time at work (less than 1 h), which is similar to previous studies: every 2 h of mean PC use was associated with a 2.23‐fold increased prevalence of TMDs,^9^ and a PC task of 4 h or longer was associated with a significantly increased prevalence of TMDs.^17^ The use of PCs for many years also affects the prevalence of TMDs.^18^

There are possible mechanisms underlying the association between VDT time and the prevalence of TMD. Previous studies have shown that depressed mood, chronic fatigue, TCH, muscular fatigue, and pain in the orofacial jaw at waking were significant factors contributing to the risk of TMDs.^19^, ^20^, ^21^ Many studies have reported that habits such as bruxism and clenching are associated with TMDs.^22^, ^23^ In addition, in the present study, even after adjusting for these factors, VDT time at work seemed to have an effect on TMD. TMDs are musculoskeletal disorders because they present with symptoms in the temporomandibular joint and masticatory muscles. VDT tasks have been shown to present symptoms at the temporomandibular joint, headache, neck pain, shoulder pain, and masticatory muscle pain,^24^ and it is reasonable to believe that VDT tasks also contribute to temporomandibular joint symptoms. Horowitz and Sarkin^3^ proposed that VDT tasks such as PC use can lead to three types of sympathetic nervous system stimulants: (a) electrostatic ambiental anion deprivation, (b) electromagnetic radiation, and (c) asthenopia and postural stress associated with poor work habits and inadequate workstation design. Many studies have examined the correlation between musculoskeletal symptoms and VDT. A previous study reported that daily VDT exposure of greater than 3 h was associated with a higher likelihood of developing physical symptoms such as headaches, neck pain, back pain, and eye exhaustion, and restricting VDT use to less than 5 h/day could prevent mental and sleep disturbances.^5^ In addition, long‐term daily uninterrupted use of VDT results in ocular fatigue and musculoskeletal pain, both of which affect mental health deterioration.^25^ On the other hand, in VDT time outside of work, the sympathetic nervous system stimulants mentioned above do not work because it is possible to work in a relaxed state, and TMD prevalence may not be increased by it. It will be important to warn people engaged in long‐term VDT at work about the risk of TMDs. The same analyses (chi‐square test and logistic regression analysis) as in the present study were performed for the quartiles of total VDT time for VDT time at work and outside of work combined, but no significant differences were found.

The present study is novel in that it investigated the effect of VDT time at work and VDT time outside of work on TMD separately and adjusted for known risk factors for TMD. Another novelty of this study is that, unlike previous studies, the subjects covered all occupational categories. However, this study had several limitations. This study was a questionnaire‐based internet survey that did not directly diagnose TMDs through dental checkups. Accurate diagnosis of TMDs requires a differential diagnosis from other diseases, along with assessing medical history and laboratory tests, such as the evaluation of tenderness and mouth opening. However, although these diagnostic criteria are applicable in the clinic, they are challenging to implement in the general population. The SQ‐TMD questionnaire used in this study has already been evaluated for its validity with TMDs and is widely used internationally.^10^ Furthermore, since the sampling of subjects was based on an Internet survey, they may differ from the general workforce. However, the proportion of high‐TRS in this study does not differ significantly from the proportion among actual workers, and it is considered that the representativeness of workers is secured to some extent. In addition, the subjects of this study were collected from all 11 occupational classifications of the Ministry of Health, Labour and Welfare (MHLW) in equal numbers of men and women. Therefore, it covers all occupational categories in Japan, and its external validity as a study to investigate the relationship between TMD and VDT time among Japanese workers is considered to be secured. In addition, the present study examined the length of VDT time, but not the type of task during VDT; the type of task during VDT may have an impact on various factors such as posture and concentration. Further detailed investigation of the content of tasks during VDT is considered necessary. In addition, since this study was a cross‐sectional survey, longitudinal studies are needed to confirm the temporal relationships.

## 5 CONCLUSIONS

This study suggests that VDT time at work influences the prevalence of TMD. In contrast, VDT time outside of work was not associated with the prevalence of TMD. Since the association between VDT time at work and the prevalence of TMD was found even after adjusting for sociopsychological factors and habits generally associated with TMD, further investigation of other factors is needed.

### ETHICAL CONSIDERATIONS

This study was conducted with the approval of the Ethics Review Board of the Faculty of Dentistry, Tokyo Medical and Dental University (D2015‐526).

## AUTHOR CONTRIBUTIONS

T.Z. and A.N. conceived the ideas; T.Z., A.O., and Y.K. collected the data; T.Z., Y.I., and J.A. analyzed the data; and T.Z. and J.A. led the writing.

## CONFLICT OF INTEREST

The authors declare that they have no conflicts of interest.

## Data Availability

The data that support the findings of this study are available from the corresponding author upon reasonable request.
